# GeneXpert—A Game-Changer for Tuberculosis Control?

**DOI:** 10.1371/journal.pmed.1001064

**Published:** 2011-07-26

**Authors:** Carlton A. Evans

**Affiliations:** 1 Universidad Peruana Cayetano Heredia, Lima, Peru; 2 Imperial College London Hammersmith Hospital Campus, London, United Kingdom; 3 IFHAD: Innovation For Health And Development, London, United Kingdom

## Abstract

Carlton Evans considers whether the new tuberculosis diagnostic test, GeneXpert, is the solution for TB control that it's said to be.

**Linked Articles**

This Perspective discusses the following new articles published in *PLoS Medicine*:

1. Scott LE, McCarthy K, Gous N, Nduna M, Van Rie A, et al. (2011) Comparison of Xpert MTB/RIF with Other Nucleic Acid Technologies for Diagnosing Pulmonary Tuberculosis in a High HIV Prevalence Setting: A Prospective Study. PLoS Med 8(7): e1001061. doi:10.1371/journal.pmed.1001061

In this prospective, real world cohort study nested within a national screening program for tuberculosis, Lesley Scott and colleagues compare the performance of Xpert MTB/RIF on a single sputum sample with different TB sputum detection technologies.

2. Lawn SD, Brooks SV, Kranzer K, Nicol M, Whitelaw A, et al. (2011) Screening for HIV-Associated Tuberculosis and Rifampicin Resistance before Antiretroviral Therapy Using the Xpert MTB/RIF Assay: A Prospective Study. PLoS Med 8(7): e1001067. doi:10.1371/journal.pmed.1001067

In a prospective study, Stephen Lawn and colleagues find that pre-ART screening with Xpert MTB/RIF increased tuberculosis case detection by 45% compared to smear microscopy in HIV-positive patients at high risk of TB risk.

3. Dowdy DW, Cattamanchi A, Steingart KR, Pai M (2011) Is Scale-Up Worth It? Challenges in Economic Analysis of Diagnostic Tests for Tuberculosis. PLoS Med 8(7): e1001063. doi:10.1371/journal.pmed.1001063

David Dowdy and colleagues discuss the complexities of costing new TB diagnostic tests, including GeneXpert, and argue that flexible analytic tools are needed for decision-makers to adapt large-sample cost-effectiveness data to local conditions.

Tuberculosis (TB) kills more people than any other single infection, the global burden of TB cases and drug resistance are increasing [1], and most patients still only have access to an inadequate diagnostic test developed more than a century ago. Recent evaluations of a desktop machine called the GeneXpert MTB/RIF that in less than two hours simultaneously detects *Mycobacterium tuberculosis* and tests for drug resistance have stimulated tremendous enthusiasm [2],[3]. Is this the breakthrough that TB control has been waiting for?

## The Backstory

TB has non-specific clinical features, so diagnosis usually requires laboratory testing. Traditional sputum smear microscopy is the only laboratory test for TB that is accessible to most of the world's population. Smear microscopy is inexpensive, appropriate for basic laboratories, rapidly diagnoses the most infectious patients, and has high specificity, so positive results almost always prompt treatment. However, smear microscopy has two key inadequacies: (1) it is insensitive, prone to false-negative “smear-negative TB” results; and (2) it cannot test for drug resistance, which is important because patients with drug-resistant TB require prompt second-line treatment to prevent morbidity, mortality, and dissemination of increasingly resistant multi-drug-resistant tuberculosis (MDRTB) and extensively drug-resistant tuberculosis (XDRTB) [4],[5]. Traditional TB culture for diagnosing smear-negative TB and testing for drug resistance takes weeks, too slow to adequately address these inadequacies. Newer rapid tests for TB and drug resistance such as MODS, Griess, MGIT, thin-layer agar, colorimetric assays, and some molecular tests [6],[7] are potential solutions but require specialised laboratories and skills that are often unavailable in the regions where most cases of TB and MDRTB occur [1].

## The GeneXpert MTB/RIF Test

The MTB/RIF test offers a potential solution for improving TB diagnosis [8]. Molecular testing enables speed, and the MTB/RIF test is feasible for use in peripheral labs and clinics by unskilled personnel [9]. In two multi-centre studies, a single MTB/RIF test detected almost all smear-positive TB patients and about three-quarters of the smear-negative TB patients whilst concurrently testing for rifampicin resistance, thus identifying patients who need second-line drug treatment [2],[3]. By enabling TB diagnosis and drug resistance testing almost anywhere without requiring the specialised laboratories and technicians needed for other rapid tests [9], this new MTB/RIF test has the capacity to be a “game-changer” in TB diagnosis.

## New Research Sounds Caution

In this week's issue of *PLoS Medicine*, three new articles raise important points of concern as the field progresses to implementation of this innovative technology. An Achilles heel of polymerase chain reaction tests for diagnosing TB is cross-contamination, in which the products from previous assays cause false-positive results. By using sealed disposable cartridges, this new MTB/RIF test apparently overcomes this problem [2],[3],[8],[10]. However, the MTB/RIF test has intermediate sensitivity, better than smear microscopy but less than broth-culture, risking false-negative results [2],[3],[8],[10]. Stephen Lawn and colleagues [11] report in this issue of *PLoS Medicine* that a single MTB/RIF test detected less than half of the cases of smear-negative culture-positive TB in HIV-positive patients being screened for TB to check whether they could safely be provided with chemoprophylaxis. Thus, a single MTB/RIF assay may be insufficient for “ruling-out” TB, although a second test for each patient increased sensitivity to 62%. Furthermore, in this week's issue, Lesley Scott and colleagues [12] and Stephen Lawn's group [11] separately report that the MTB/RIF assay occasionally provides incorrect assessments of rifampicin resistance, principally false resistance, as has been reported previously. Whilst these errors are uncommon, they are inevitable because the speed of genotypic testing for TB drug resistance is a valuable but imperfect surrogate for the slower phenotypic culture-based tests. David Dowdy and colleagues, in a third paper in this week's *PLoS Medicine* [13], add to the Xpert story by providing new insights into the complexities of comparing the benefits of increased correct diagnoses with the adverse consequences of occasional misdiagnoses, concluding that standard cost-effectiveness analyses may give misleading results.

## What about Accuracy?

The MTB/RIF test enables TB detection and rifampicin resistance testing near the point of care, facilitating rapid screening for TB and drug resistance. However, the accuracy issues described above suggest that access to confirmatory culture-based testing will still be required in many settings. Rifampicin-resistant TB is usually MDRTB and always requires second-line drug therapy, so this has immediate treatment implications [3],[5]. Patients found by the MTB/RIF test to have rifampicin-resistant TB still require specialist laboratory facilities for more extensive drug resistance testing. Paradoxically, the benefit of MTB/RIF test implementation through identifying more rifampicin-resistant TB is likely to actually increase the demand for specialist reference laboratories to indicate how those patients infected with rifampicin-resistant TB should be treated.

## Limitations of the Test

The MTB/RIF test is a major advance in TB diagnostic testing, but has limitations, including the limited shelf-life of the diagnostic cartridges, some operating temperature and humidity restrictions, requirement for electricity supply, unknown long-term robustness, and the need for annual servicing and calibration of each machine [14]. Laboratories in low-income countries are littered with expensive equipment that no longer functions because it was inappropriate to the setting to which it was donated. Ensuring sustainable systems for long-term provision of servicing and consumables may be more important and challenging than initial implementation of the diagnostic equipment itself.

## Impact in Low- and Middle-Income Settings

While effectiveness analysis and feasibility studies are necessary, they are poor surrogates for predicting the impact of the MTB/RIF test in programmatic use [15]. More broadly, lab-based accuracy data are not sufficient to judge the contribution of new diagnostic tools for case finding, treatment, cure, and ultimately TB control. Despite numerous microbiological studies of improved TB diagnostic technologies, we remain remarkably ignorant of how best to implement better tests to improve patient care, of who should receive the limited capacity for better tests to maximise health impact, of how these tests may impact patient-relevant outcomes, and of how these issues vary between settings [15]. The impact of better diagnostic tests on the equity of care is largely unstudied and we don't know yet how this novel technology will affect the delays and costs faced by patients in their journey towards a cure for this archetypal disease of poverty.

## The Greatest Challenge for GeneXpert: Case-Finding

In 2009, only 63% of all TB cases were estimated to have been diagnosed, partly because smear microscopy is so insensitive that it fails to detect TB in a third of patients who would be diagnosed by culture [1]. Smear microscopy is particularly insensitive for diagnosing TB in patients at high risk of dying from TB, including children and people living with HIV [9]. However, providing TB tests that are more sensitive than smear microscopy is confounded by the non-specific nature of TB symptoms—most people tested for TB are negative by all TB tests because they actually have other diseases, not TB [16]. For example, in a Peruvian community, one in 14 individuals tested for suspected TB had positive smear microscopy [17]. In contrast, in the same community, each MTB/RIF test diagnosed TB in one in 34 of the selected patients with suspected TB who were smear-negative [2]. Such statistics assessing the number of tests required to impact upon each patient-important outcome are key to the optimal implementation of TB diagnostics, but are rarely reported by research studies [15]. This damaging omission must be corrected in future research. As with any TB test, many people will have to be tested for each smear-negative individual with suspected TB diagnosed [2]. Consequently in some settings, providing the relatively expensive MTB/RIF test for TB case-finding may only be sustainably affordable for selected patient groups and may be less cost-effective than behavioural interventions [18] or focusing the provision of this test for drug resistance testing.

## The Ongoing Toll of Drug Resistance

TB drug resistance prevalence is increasing [1], and of the estimated 500,000 people annually who develop MDRTB, less than 7% are diagnosed and only one in five of these receive effective treatment [19]. The introduction of a new diagnostic test, no matter how good, doesn't necessarily imply clinical benefit because better TB tests only lead to better health if populations can afford to access them and act effectively upon their results. Indeed, studies frequently report success diagnosing MDRTB and XDRTB cases that then remain untreated [20]–[22] despite the demonstrated achievability of effective MDRTB care [23],[24]. Clearly, the current widespread failure to adequately manage the great majority of the MDRTB that is already diagnosed is no justification for failing to diagnose the rest. To be sure, increasing universal rapid MDRTB diagnosis is important for meeting the human rights and public health needs for universal access to MDRTB treatment and the MTB/RIF test has the capacity to greatly facilitate this process. However, six-months curative treatment for a TB patient costs a few tens to a few hundreds of dollars, but MDRTB treatment costs ten to a hundred times more, several thousands of dollars [25]. Thus, in many settings, the costs of MTB/RIF testing are likely to be dwarfed by the cost of treating the drug-resistant TB that it will diagnose.

Furthermore, MDRTB management requires skills and specialist drug supply that currently have severely restricted availability in the low-income countries where most MDRTB occurs [1]. Consequently, the greatest challenges concerning MTB/RIF screening of new TB patients for drug resistance will likely be to ensure rapidly expanded capacity to manage drug-resistant TB. The early diagnosis of drug-resistant TB can be cost-effective [25], but risk factors for TB drug resistance have poor predictive value, especially in high-prevalence settings [26], so universal drug resistance testing of all new TB patients by the MTB/RIF or other rapid tests is a priority in many settings. Despite these challenges, the global public health community will be wise to take the opportunity offered by rapid MDRTB tests, including the MTB/RIF test, to urgently invest in preventing the global increase in drug-resistant TB.

## Cost Will Always Be Key

An important limitation of the MTB/RIF test is its cost, which may be prohibitive for a disease that principally affects poor people in poor communities [9]. With tiered pricing for low-income countries, each MTB/RIF test machine currently costs US$17,000–$62,000. More importantly, each disposable test-cartridge costs US$17–$120 [27], which is comparable with the per capita annual health expenditure in the countries with the highest TB burdens. Although much more expensive than smear microscopy, affordability varies greatly between settings, these costs are expected to fall, and they appear to be comparable with the total costs of providing other rapid TB tests [27]. Furthermore, experiences with HIV viral load and CD4 cell counting tests have demonstrated that advocacy can convince donors to fund the rapid implementation of relatively expensive diagnostic technologies when needs and benefits are clear, and the MTB/RIF test may be such a test. Thus, costliness may not prevent the roll-out of this test to the limited numbers of TB patients requiring drug resistance testing, but may severely restrict the availability of this assay for the much larger numbers of people needing testing for suspected TB. Despite the fact that funding for TB control in high-burden countries has more than doubled between 2002 and 2009, large funding gaps remain, and many countries are currently struggling to sustain basic diagnostic and treatment services. For example, it has been estimated that in India, providing the MTB/RIF test to only 15% of the suspected cases of TB would consume the annual budget for the entire TB control program [13].

## Conclusion

The MTB/RIF test should make rapid drug resistance testing more widely achievable and, in selected groups, may strengthen TB case finding. Its impact will inevitably be limited by its expense, but it may be cost-effective and useful for rapidly screening TB patients for drug resistance if funding for increased MDR TB treatment also becomes available. Whilst this advance should be celebrated and funding for it should be prioritised, this must be viewed within the shameful context that almost 2 million people die each year from TB, and very few of them would have been saved by any diagnostic test. Specifically, these deaths occur in mainly HIV-negative people, almost all of whom die from drug-susceptible TB, principally because of the inadequacy of basic, inexpensive health care provision for this curable infectious disease. Funding should be identified to make the MTB/RIF test available in suitable settings, but the current financial crisis coupled with the huge unmet needs in other health areas will make the competition for resources even more intense [28]. This new test must not divert resources from preventive efforts and well-established TB diagnostic and treatment systems that already have the potential to have considerable impact upon TB morbidity and mortality.

TB control programs have “averted millions of deaths, but their effects on TB transmission and incidence rates are not yet widely detectable” [29]. Poverty and social factors remain the principal determinants of global TB rates, not TB control efforts [29],[30]. There is increasing consensus that tools such as the new MTB/RIF test must be integrated with interventions that address the socioeconomic determinants of TB [28],[31]–[33] if they are to help current achievements in TB care to result in TB control.

## Abbreviations

MDRTB: multi-drug-resistant tuberculosis
TB: tuberculosis
